# Supplementary material for: High prevalence of antimicrobial resistance and multidrug resistance among bacterial isolates from diseased pets: Retrospective laboratory data (2015–2017)
Source: PLoS One. 2022 Dec 7;17(12):e0277664. doi: 10.1371/journal.pone.0277664 (PMC9728933; doi:10.1371/journal.pone.0277664)
Supplement: Attachment — Submitted filename: Reviewer_PlosOne_ao.pdf [file pone.0277664.s001.pdf]

July 18, 2022

Dr. Emmanuel Serrano, PhD
Editor – PLos One

Dear Editor,

RE: High Prevalence of Antimicrobial Resistance and Multidrug Resistance Among Bacterial Isolates from Diseased Pets: Retrospective Laboratory Data (2015-2017)

Thank you very much indeed for your email dated on June 21, 2022 and the invitation to review a Manuscript with ref. No: PONE-D-22-09971. I have read the manuscript and feel that the paper might improve as a result of the peer review process.

- Please find my comments and suggestions to the authors and hope these would be addressed accordingly

Sincerely,

Abdinasir Yusuf Osman, PhD
The Royal Veterinary College, University of London, UK
E: aosman@rvc.ac.uk

***

GENERAL COMMENTS

- Overall, this is an interesting and timely paper on the evidence on the emerging Antimicrobial Resistance in companion animals in Malaysia.
- That being said there are several areas for clarification and elaboration that would strengthen the study.

- **Scope:**
    - Although the aim of the paper was to analyze a retrospective data between 2015 to 2017 among companion animals presented to veterinary hospital, a significant gap is the lack of inclusion of data between 2017- 2022 missing accurate information on the current picture of AMR in the target population.

- Given that the prevalence of AMR is highly likely to be low in household-based setting compared to hospital setting as the case here, the authors need to clarify why this pertinent data was excluded.

- Related to the veracity of the confounding factors in some circumstances, the reported prevalence may not be specific, hence the lower proportion of these than expected. To establish this as evidence, the authors might make a correlation between antimicrobial use (AMU) in companions animals vs AMR to identify pathways of the drivers of AMR in Malaysia. Comparison of AMR between different settings (hospital as the case here vs household would further strengthen the impact of the paper.

Abstract

- **Rationale**
  - My concern is that the brief of the rationale and the existing gaps has NOT been be briefly described.
  - The authors presented retrospective data retrieved between 2015 – 2017 and up to date data missing great proportion of the totality of the evidence after 2017 [5 years].

MATERIALS & METHODS

Source of the data

- The appended below sentence (see line 90-94) should be shifted to results section as it is not related to the methodology.
  - *Fig 1 shows the distribution and percentages of bacterial species isolated during the study period. Escherichia coli was the most common isolate for Gram-negative bacteria followed by Proteus mirabilis and Klebsiella, whereas for Gram-positive bacteria, Staphylococcus pseudintermedius was the most common isolate followed by Streptococcus canis and Staphylococcus intermedius.*

- Please clarify the following sentence:
  - 'For the purpose of this analysis, the isolates categorized as 'intermediate' and 'resistant' were grouped as 'non-susceptible'.

- **RESULTS**
  - Please define the acronym used in the fig 2-11. Please apply this change throughout.
  - In Table 1: samples [postmortem, reproductive, urinary tract], please specify the nature of sample, and type of the organs rather than categorizing them as system-based classification.
  - Please add a footnote to the table 1 and introduce the [others], etc.
  - Please write as Fig 2-5 in line 145 and Table 3, 4 in line 293

- Rectify the footnote of Table 4; [*NA- should be written NOT Analyzed rather than Not Included to avoid confusion(see line 317).

- **DISCUSSION**
  - The discussion section, even though should NOT be sub structured, remains superficial and not country specific. Comparisons are often made at global scale rather than regional and national levels missing crucial information about the epidemiology of AMR in the context of current research in Malaysia and the wider Southeast Asia.

  - The author (s) should also discuss the strength of their study and how their manuscript contribute to the literature and fit within the context of current research or practice.
  -
  - The author (s) should also discuss the limitation of their study of using admitted cases as these that will overestimate of the prevalence of AMR, thus the call for a nationwide AMR prevalence study.

End*
