# Supplementary material for: High prevalence of antimicrobial resistance and multidrug resistance among bacterial isolates from diseased pets: Retrospective laboratory data (2015–2017)
Source: PLoS One. 2022 Dec 7;17(12):e0277664. doi: 10.1371/journal.pone.0277664 (PMC9728933; doi:10.1371/journal.pone.0277664)
Supplement: Attachment — Submitted filename: List Of Correction PLOS ONE_Final.docx [file pone.0277664.s002.docx]

**Title: High Prevalence of Antimicrobial Resistance and Multidrug Resistance among Bacterial Isolates from Diseased Pets: Retrospective Laboratory Data (2015-2017)**

Dear Reviewer,

We appreciate the constructive comments from the reviewer and believe that these comments have improved the overall quality and readability of the manuscript. Below are the point-by-point responses to the comments of the reviewer. We sincerely hope that this manuscript is now more acceptable for further review and publication in this journal.

| **NO.** | **COMMENTS FROM THE REVIEWER** | **RESPONSE AND CORRECTIONS MADE** |
| --- | --- | --- |
|  | **A. SCOPE** |  |
| 1. | Although the aim of the paper was to analyze a retrospective data between 2015 to 2017 among companion animals presented to veterinary hospital, a significant gap is the lack of inclusion of data between 2017-2022 missing accurate information on the current picture of AMR in the target population. | This project was part of a Master research project which started in 2018, therefore was scoped for the previous three-year retrospective lab report (2015-2017). We appreciate the suggestion of expanding the period of study however given the timeline of the Master’s programme, this was not possible.  However, since almost no information exists in the country and very few publications exists regionally or globally on the AMR of clinically important pathogens of small animals, we believe that this study has provided significant information on the overall picture of resistance pattern of pathogens that are important in causing illnesses in cats and dogs giving insight about the local trends of AMR at the national, regional, and global level. |
| 2. | Given that the prevalence of AMR is highly likely to be low in household-based setting compared to hospital setting as the case here, the authors need to clarify why this pertinent data was excluded. | The overall aim of this research is to address the knowledge gap and describe the situation of the AMR among clinical isolates through a retrospective examination of available diagnostic data. Most cases presented were from veterinary health premises and animal facilities received by the accredited diagnostic bacteriology laboratory at the university. We have made it explicit that data from this study does not necessarily represent the prevalence of AMR from pets in households or general cats and dogs populations in Peninsular Malaysia. |
| 3. | Related to the veracity of the confounding factors in some circumstances, the reported prevalence may not be specific, hence the lower proportion of these than expected. To establish this as evidence, the authors might make a correlation between antimicrobial use (AMU) in companions animals vs AMR to identify pathways of the drivers of AMR in Malaysia. Comparison of AMR between different settings (hospital as the case here vs household would further strengthen the impact of the paper. | Thank you for the comment. We fully recognized and acknowledged the inherent limitations of our findings and have addressed this in the original manuscript (Page 27). Unfortunately, the samples received by the diagnostic laboratory from pets were collected from diseased animals with incomplete history of antibiotic treatment(s). Thus, we cannot correlate between antimicrobial use (AMU) and antimicrobial resistance (AMR). In addition, as with many parts of the world, but especially reflective of the local and regional situation at the time of the study and remained true at present, the use of antibiotics in animals for both livestock and pets are poorly monitored. Monitoring and quantifying AMU is one of the strategy for the national AMR action plan for Malaysia that has been extended into the next action plan 2022-2027 due to the complexity and challenges of acquiring this type of information. |
|  | **B. ABSTRACT (Rationale)** |  |
| 4. | My concern is that the brief of the rationale and the existing gaps has NOT been being briefly described. | See No. 1 |
| 5. | The authors presented retrospective data retrieved between 2015 – 2017 and up to date data missing great proportion of the totality of the evidence after 2017 [5 years]. | See No. 1 |
|  | **C. MATERIALS AND METHODS (Source of the data)** |  |
| 6. | The appended below sentence (see line 90-94) should be shifted to results section as it is not related to the methodology.   - *Fig 1 shows the distribution … followed by Streptococcus canis and Staphylococcus intermedius*. | Figure 1 has been shifted to the Results section in page 7. |
|  | Please clarify the following sentence:   - ‘For the purpose of this analysis, the isolates categorized as ‘intermediate’ and ‘resistant’ were grouped as ‘non-susceptible’. | For the purpose of statistical analysis (Chi-square test), those isolates categorized as ‘intermediate’ from antibiotic susceptibility testing were not excluded from the study, instead were combined and grouped together with ‘resistant’ and named ‘non-susceptible’. |
|  | **D. RESULTS** |  |
|  |  |  |
| 7. | Please define the acronym used in the fig 2-11. Please apply this change throughout. | The acronyms were readily presented in the original manuscript. |
| 8. | In Table 1: samples [postmortem, reproductive, urinary tract], please specify the nature of sample, and type of the organs rather than categorizing them as system based classification. | The samples of the diseased animals presented to the bacteriology laboratory from various types of samples were combined and categorized as system-based increase data robustness for analysis because the number of isolates per type of samples varies widely and can be low. |
| 9. | Please add a footnote to the table 1 and introduce the [others], etc. | Footnote for table 1 and 2 has been added in page 9 and 14 |
| 10. | Please write as Fig 2-5 in line 145 and Table 3, 4 in line 293. | Corrected in page 9 and 18 |
| 11. | Rectify the footnote of Table 4; [*NA- should be written NOT Analyzed rather than Not Included to avoid confusion (see line 317). | Corrected in page 19 and 20 |
|  | **E. DISCUSSION** |  |
| 12. | The discussion section, even though should NOT be sub structured, remains superficial and not country specific. Comparisons are often made at global scaler rather than regional and national levels missing crucial information about the epidemiology of AMR in the context of current research in Malaysia and the wider Southeast Asia. | Thank you for the comment and we acknowledge the limitation of available literature to discuss the findings of this study. Research and publication of AMR in the veterinary context and especially focusing on pathogens in pets is not extensive in Malaysia and in the region. We have addressed this issue in the Introduction and added a few lines in the Discussion (Page 21 Linen 383-385).  In Malaysia, there is only one publication by Shahaza et al*.,* [79] which was included in the discussion part of this manuscript, that presented the analysis of *E. coli* of clinical isolates from various species. There is no publication about clinical isolates in pets at the regional level which was why most of the references and comparisons were from more extended populations of pets in Europe, Canada and Australia. |
| 13. | The author (s) should also discuss the strength of their study and how their manuscript contribute to the literature and fit within the context of current research or practice. | The strength of this study was discussed in Introduction, page 4, line 63 to 68. |
| 14. | The author (s) should also discuss the limitation of their study of using admitted cases as these that will overestimate of the prevalence of AMR, thus the call for a nationwide AMR prevalence study. | The limitation of the study was discussed in page 27. |
