# Supplementary material for: High prevalence of antimicrobial resistance and multidrug resistance among bacterial isolates from diseased pets: Retrospective laboratory data (2015–2017)
Source: PLoS One. 2022 Dec 7;17(12):e0277664. doi: 10.1371/journal.pone.0277664 (PMC9728933; doi:10.1371/journal.pone.0277664)
Supplement: Attachment — Submitted filename: List Of Correction PLOS ONE_Second Revision.docx [file pone.0277664.s003.docx]

**Title: High Prevalence of Antimicrobial Resistance and Multidrug Resistance among Bacterial Isolates from Diseased Pets: Retrospective Laboratory Data (2015-2017)**

Dear Reviewer,

We appreciate the constructive comments from the reviewer and believe that these comments have improved the overall quality and readability of the manuscript. Below are the point-by-point responses to the comments of the reviewer. We sincerely hope that this manuscript is now more acceptable for further review and publication in this journal.

| **NO.** | **COMMENTS FROM THE REVIEWER** | **RESPONSE AND CORRECTIONS MADE** |
| --- | --- | --- |
|  | **A. REFERENCES** |  |
| 1. | Please review your reference list to ensure that it is complete and correct. If you have cited papers that have been retracted, please include the rationale for doing so in the manuscript text, or remove these references and replace them with relevant current references. Any changes to the reference list should be mentioned in the rebuttal letter that accompanies your revised manuscript. If you need to cite a retracted article, indicate the article’s retracted status in the References list and also include a citation and full reference for the retraction notice. | 1) In text citation for:   - [1] (Page 2, Line 35) - [1] (Page 26, Line 464) - [22–24] (Page 4, Line 63) - [1,74] (Page 25, Line 437) **has been updated to the latest version.**   2) Reference for:  24. Moyaert H, de Jong A, Simjee S, Rose M, Youala M, El Garch F, et al. Survey of antimicrobial susceptibility of bacterial pathogens isolated from dogs and cats with respiratory tract infections in Europe: ComPath results. J. Appl. Microbiol. 2019 April; 127(1): 29-46. <https://doi.org/10.1111/jam.14274>  **has been revised to**  24. Moyaert H, de Jong A, Simjee S, Rose M, Youala M, El Garch F, et al. Erratum: Survey of antimicrobial susceptibility of bacterial pathogens isolated from dogs and cats with respiratory tract infections in Europe: ComPath results. J. Appl. Microbiol. 2019 November; 127(5): 1594. <https://doi.org/10.1111/jam.14420>  3) Statement for:   - (Page 2, Line 33-35): ……… isolates in pets is an emerging issue of veterinary and public health significance [1] **has been revised** **to** ……… isolates such as MRSA and MDR *E. coli* and *Klebsiella pneumoniae* in pets is an emerging issue of veterinary and public health significance [1]. - (Page 6, Line 114-116): ……… as ‘multi-drug resistant’ if it were resistant to at least three or more classes of antibiotics [36] **has been revised** **to** ……… as ‘multi-drug resistant’ if it were resistant to at least one agent in three or more classes of antibiotics [36]. - (Page 25, Line 436-437): ……… wide range of pets and as part of the normal microbiota of pets [1,74] **has been revised to** ……… wide range of companion animals and as part of the their normal microbiota [1,74]. - (Page 26, Line 463-464): ……… between pets and humans and this creates opportunities for interspecies transmission of AMR-bacteria [1] **has been revised** **to** ……… between companion animals and humans and this creates opportunities for interspecies transmission of AMR-bacteria [1]. |
|  | **B. FIGURES** |  |
| 2. | The presented figures as a whole show poor quality and thus re-plotting probably using more sophisticated software to enhance their quality is highly recommended | Figures have been updated to the good quality using Preflight Analysis and Conversion Engine (PACE) digital diagnostic tool. |
|  | **C. STRENGTH OF THE STUDY** |  |
| 3. | The strength of the study should be written [re-highlighted] in the discussion section. | The strength of the study has been highlighted in the discussion section (Page 20-21, Line 343-350) |
